# Delayed diagnosis of a transient ischemic attack caused by ChatGPT

**DOI:** 10.1007/s00508-024-02329-1

**Published:** 2024-02-02

**Authors:** Jonathan A. Saenger, Jonathan Hunger, Andreas Boss, Johannes Richter

**Affiliations:** 1 Diagnostic and interventional Radiology, University Hospital Zurich, University Zurich, Zurich, Switzerland; 2 Institute of Radiology and Nuclear Medicine, GZO Hospital Wetzikon, Wetzikon, Switzerland; 3 Department of Internal Medicine, GZO Hospital Wetzikon, Wetzikon, Switzerland; 4 Neurology and Stroke Unit, GZO Hospital Wetzikon, Wetzikon, Switzerland

**Keywords:** AI, Stroke, ChatGPT, Neurology, Chatbot, Artificial Intelligence

## Abstract

Techniques of artificial intelligence (AI) are increasingly used in the treatment of patients, such as providing a diagnosis in radiological imaging, improving workflow by triaging patients or providing an expert opinion based on clinical symptoms; however, such AI techniques also hold intrinsic risks as AI algorithms may point in the wrong direction and constitute a black box without explaining the reason for the decision-making process.

This article outlines a case where an erroneous ChatGPT diagnosis, relied upon by the patient to evaluate symptoms, led to a significant treatment delay and a potentially life-threatening situation. With this case, we would like to point out the typical risks posed by the widespread application of AI tools not intended for medical decision-making.

## Background

Patients consulting online sources to gather information about medical symptoms is a current and widespread phenomenon in health information-seeking behavior [1, 2]. The latest step in this process was the publication of the Artificial Intelligence (AI) powered Chat Generative Pre-trained Transformer (ChatGPT) (OpenAI, San Francisco, CA, USA) on 30 November 2022. Here, we present the case of a 63-year-old man with neurological symptoms whose diagnosis of transient ischemic attac (TIA) was delayed due to an incomplete diagnosis and interpretation by ChatGPT.

## Case report

A man in his 60s presented to the emergency department (ER) of our hospital with a history of multiple self-limiting episodes of diplopia, having undergone pulmonary vein isolation (PVI) for atrial fibrillation 4 days earlier. Even though he was adherent to the prescribed medication of rivaroxaban 20 mg, he was concerned that his symptoms might be complications of the procedure. He had contacted his interventionalist, who had classified the symptoms as harmless after-effects of PVI. If the symptoms recurred, he was advised to contact a general practitioner or to go to the ER. Not finding the interventionalist’s explanation satisfactory, he consulted ChatGPT model 3.5 at the second onset of symptoms. Although he was already considering stroke as a possible explanation for his symptoms, he hoped ChatGPT would provide a less severe explanation to save him a trip to the ER. Relieved that ChatGPT had also classified “vision problems” as “possible” after PVI, the patient decided to stay at home. When confronted later, he described the doctors’ explanation as “partly incomprehensible” and the chatbot’s answer as a “valuable, precise and understandable risk assessment”.

The patient decided to call an ambulance approximately 24 h later when a third episode occurred. The clinical examination in the ER was unremarkable. The neurological examination by an attending neurologist remained unremarkable except for paresthesia, hyposensitivity, and fine motor disturbance of the left hand, which equals one point on the National Institutes of Health Stroke Scale (NIHSS). Due to the medical history and the persistent neurological deficits, acute stroke remained the working diagnosis. Emergency computer tomography with angiography (CTA) and subsequent magnetic resonance imaging (MRI) the morning after admission showed no signs of acute infarction. Initial electrocardiography (ECG) showed a normocard sinus rhythm; however, the ECG monitoring reported a recurrence of paroxysmic atrial fibrillation. Subsequent work-up remained unremarkable except for a previously unknown patent foramen ovale. The patient was admitted to the stroke unit, where all neurological deficits wholly resolved within 1h. Therefore, the working diagnosis was changed to TIA. Due to risk factors and the fact that the TIA occurred even though the patient was taking rivaroxaban, the anticoagulant was switched to apixaban two times a day 5 mg, and clopidogrel 75 mg, metoprolol 25 mg and atorvastatin 40 mg were added. The patient was discharged home after 2 days without residual neurological deficits.

## Discussion

This case underscores the potential of AI as a valuable tool but also highlights the risks associated with blind reliance on it. Although not specifically designed for medical advice, ChatGPT answered all questions to the patient’s satisfaction, unlike the physician, which may be attributable to satisfaction bias, as the patient was relieved by ChatGPT’s appeasing answers and did not seek further clarification. Rephrasing the patient’s questions by directly asking ChatGPT if visual impairment following catheter ablation could indicate a stroke, would have resulted in an affirmative response. Interestingly, the same effect was observed with Google Bard (Google LLC, Mountain View, CA, USA). As documented, ChatGPT can provide inconsistent and inappropriate recommendations [3] and fabricate data to support prior claims [3]. Furthermore, ChatGPT’s training data were limited to September 2021 and may be outdated and unaware of recent medical advancements, making it susceptible to errors.

Unmoderated communication with a chatbot may yield less than optimal results, as patients may need help to discern which symptoms are relevant to the diagnostic process. As demonstrated in Tab. 1 rephrasing the questions could have drastically changed the Chatbot’s response. Due to its risk-averse nature, the chatbot may struggle to exclude certain diagnoses when presented with nonspecific symptoms, potentially leading to an increased burden on the healthcare system by directing more patients to hospitals [4] or causing patients to stay home when medical intervention would be necessary [5]. When evaluated through established principles of medical ethics, it remains to be seen if potential benefits of expediting a diagnosis through AI outweigh the risk of erroneous recommendations.

Therefore, chatbots may prove more valuable on the healthcare professional’s side, having the potential to alleviate the burden on overworked professionals [6], reduce the risk of errors [7] and offer predictive analysis [8]. The legal and ethical implications hereof still need to be addressed by regulatory bodies and discussed by society at large. To our knowledge, the legally protected doctor’s confidentiality does not or does not fully apply to chatbot queries.

In an era marked by demographic shifts, workforce shortages, and increasing economic pressures on the healthcare system, AI systems and chatbots can serve as valuable additions to the healthcare environment. To ensure robust medical and scientific foundations, medical professionals must be actively involved in developing medical AI systems. The challenge for the future is to determine how AI can enhance medical consultations in a safe, effective, and ethical manner [9]. AI should complement, rather than replace healthcare professionals to ensure a safer and more effective hospital environment in the future [9].

**Table 1:** Excerpt from the patient’s chat log with ChatGPT, showcasing a comparison between the patient’s original question and the authors’ edited version. Notably, the patient’s query, particularly the final sentence expressing concerns about visual disturbances post-catheter ablation, triggered a more alarming response when input into Google BARD. This contrast became even more apparent when the authors posed the direct question, suggesting that Google BARD’s superiority may stem from its direct internet access

	Patient’s original question: Is it possible to have visual disturbance after catheter ablation?	Authors’ edited version: I have visual disturbances after catheter ablation. Could this be a stroke?
*ChatGPT*	**Yes, it is possible to experience visual disturbances after catheter ablation.** “…” Although visual disturbances are not one of the most common side effects of catheter ablation, they can occur in some cases. Visual disturbances after catheter ablation can have several causes: Medication “…”, Blood pressure changes “…” and Nerve stimulation “…”. Catheter ablation may be performed near nerve pathways and tissues associated with the eyes. In rare cases, this may cause temporary visual disturbances. **It is important that you inform your attending physician about any visual disturbances that occur after catheter ablation. **They will be able to assess your specific situation and determine if further testing or action is needed. However, in most cases, visual disturbances after catheter ablation are temporary and will improve on their own within a short period of time	**Visual disturbances after a catheter ablation procedure are not a common or expected side effect of the procedure itself.** Catheter ablation is a medical procedure used to treat certain heart rhythm disorders, and it is not directly related to visual problems. However, visual disturbances can sometimes be associated with other medical conditions or complications, including neurological issues. **If you experience sudden or severe visual disturbances after a catheter ablation, it’s important to seek immediate medical attention.** **While it’s possible that visual disturbances could be related to a stroke**, there can be other causes as well. Only a medical professional can provide a proper evaluation and diagnosis based on your specific symptoms and medical history. **Do not delay seeking medical care if you are experiencing concerning visual disturbances**, as prompt evaluation and treatment are essential to determine the underlying cause and ensure the best possible outcome
*Google BARD*	**Yes, it is possible to have visual disturbance after catheter ablation.** Visual disturbance after catheter ablation is a rare complication, but it can occur. The most common type of visual disturbance is migraine with aura. “…” Other types of visual disturbance that can occur after catheter ablation include: Blurred vision, Double vision, Loss of vision in one or both eyes, Sensitivity to light The exact cause of visual disturbance after catheter ablation is not known. However, it is thought to be related to the following factors: Inflammation of the blood vessels in the brain, Damage to the optic nerve, Microemboli (tiny blood clots) traveling to the brain. Visual disturbance after catheter ablation is usually temporary and resolves on its own within a few days or weeks. However, in some cases, it can be permanent. **If you have visual disturbance after catheter ablation, it is important to see a doctor right away to rule out any serious underlying causes**. “…” Here are some things you can do to reduce your risk of visual disturbance after catheter ablation: “…” Drink plenty of fluids to help your body flush out any contrast dye that was used during the procedure. “…”. See your doctor regularly for follow-up care. “…”	**Yes, visual disturbance after a catheter ablation could be a sign of stroke.** “…” If you have visual disturbance after a catheter ablation, it is important to see a doctor right away to rule out stroke. “…”. If you have a stroke, it is important to get treatment right away to minimize the damage to your brain. Treatment may include medications, surgery, and rehabilitation. Here are some things you can do to reduce your risk of stroke after a catheter ablation: Talk to your doctor about your risk factors for stroke and what you can do to reduce them. Follow your doctor’s instructions carefully after the procedure. Take all medications as prescribed. Drink plenty of fluids to help your body flush out any contrast dye that was used during the procedure. Avoid strenuous activity for several days after the procedure. See your doctor regularly for follow-up care. If you have any concerns about your vision or other health problems after a catheter ablation, be sure to talk to your doctor
